# Magnetic resonance imaging findings of a case with Wolffian tumor and related literature review

**DOI:** 10.2478/abm-2024-0012

**Published:** 2024-04-30

**Authors:** Can Cui, Dawei Cui, Jiangfeng Pan, Shaobin Zhou, Xiujuan Zheng

**Affiliations:** Department of Radiology, Jinhua Municipal Central Hospital (Affiliated Jinhua Hospital, Zhejiang University School of Medicine), Zhejiang 321000, China; Department of Obstetrics and Gynecology, Jinhua Municipal Central Hospital (Affiliated Jinhua Hospital, Zhejiang University School of Medicine), Jinhua Maternal and Child Health Care Hospital, Zhejiang 321000, China

**Keywords:** female adnexal tumor, magnetic resonance imaging, ovarian tumor, Wolffian tumor

## Abstract

**Background:**

Wolffian tumors in females are rare gynecological neoplasms, with fewer than 100 cases reported. Existing literature primarily focuses on the pathology, and reports involving imaging are limited.

**Objective:**

This study presents a case of Wolffian tumor, emphasizing its magnetic resonance imaging (MRI) characteristics to enhance preoperative diagnostic accuracy.

**Case report:**

A 56-year-old woman presented with a year-long history of irregular vaginal bleeding. MRI revealed a solid mass in the right adnexal region. On T2-weighted images, the mass exhibited slightly elevated signal intensity with a distinctive low-signal intensity rim. Diffusion-weighted imaging displayed markedly increased signal intensity, and the contrast enhancement was moderate. The patient underwent laparoscopic right adnexectomy and received a Wolffian tumor diagnosis. No recurrence was observed during a 6-month follow-up.

**Conclusions:**

Wolffian tumors exhibit distinctive MRI presentations. Notably, the prominent low-signal intensity rim on MRI may aid in accurate preoperative tumor diagnosis.

Wolffian tumors, rare gynecological neoplasms originating from probable Wolffian tissue, were initially described by Kariminejad and Scully [1] in 1973. Fewer than 100 cases of Wolffian tumors have been documented, with a scarcity of imaging-related reports. The present study presents magnetic resonance imaging (MRI) findings from a Wolffian tumor case and reviews relevant literature to elucidate clinicopathological features, imaging diagnostics, and differential diagnoses to enhance the understanding of MRI features.

The present study was approved by the Ethics Review Board of Jinhua Municipal Central Hospital (Affiliated Jinhua Hospital, Zhejiang University School of Medicine), Jinhua, Zhejiang, China, in accordance with the Declaration of Helsinki. Written informed consent was obtained from the patient included in the study.

## Case report

A 56-year-old postmenopausal woman was admitted to the hospital due to a 1-year history of irregular vaginal bleeding, accompanied by a pelvic mass detected via ultrasound. The patient had experienced irregular vaginal bleeding, similar to menstruation, with high volume, bright red color, and blood clots, without an obvious trigger. She had initially sought care at a local county hospital, where ultrasound identified a heterogeneous hypoechoic mass in the right adnexal region measuring approximately 10.7 cm × 10.1 cm with well-defined borders and an anechoic area of about 3.9 cm × 2.2 cm. Additionally, multiple hypoechoic nodules, roughly 5.0 cm × 3.9 cm in size, were noted in the myometrium. Following this diagnosis, the patient was admitted to our hospital for further evaluation. On admission, the patient reported vaginal bleeding resembling menstruation, without abdominal pain, distension, urinary symptoms, fever, chills, panic, or weakness. She denied a history of gynecological diseases, family history of malignancy, or hereditary syndromes.

Physical examination revealed a normal vulva with a patent vagina. A small quantity of odorless blood was observed. The cervix appeared normal, with a flat texture and smooth surface. No blood was found upon palpation, and no tenderness or pain was noted. The uterus was of normal size and anteriorly positioned. A well-defined swelling measuring approximately 10 cm × 10 cm was palpable in the right adnexal area, demonstrating a hard texture. Abdominal distension occurred upon pressure but without tenderness. The left adnexa were indistinct upon palpation. Tumor markers (CA125, CA19–9) and cancer-related indicators (carcinoembryonic antigen, methemoglobin, and serum ferritin) were within normal ranges.

## Imaging findings

**Figure 1.**
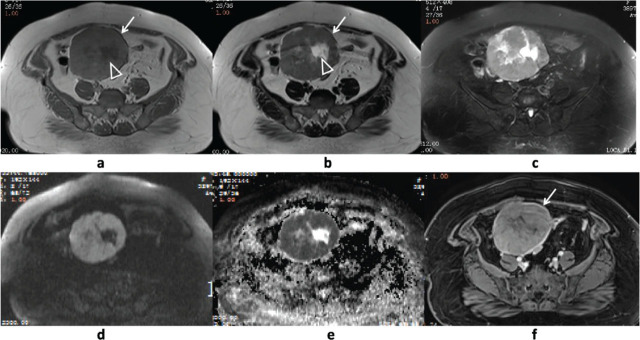
Pelvic MRI revealed a solid oval mass in the right adnexal region. It exhibited iso-signal intensity on T_1_WI **(a)**, slightly higher-signal intensityonT_2_WI (**b**, relative to muscle), and T_2_WI fat-suppressed axial images **(c)**. The mass possessed a capsule (indicated by the arrow in **a,b**) and central cystic necrosis (triangular arrow in **a,b**). The mass displayed marked high intensity on the diffusion-weighted image (B = 800 s/mm^2^) (**d**) with restricted diffusion on the ADC map (**e**). The solid part of the mass exhibited homogeneous and moderate enhancement with intravenous gadolinium-DTPA, and the envelope (arrow) also demonstrated enhancement (**f**). ADC, apparent diffusion coefficient; MRI, magnetic resonance imaging.

MRI revealed a well-defined oval solid mass measuring approximately 110 mm in diameter in the right adnexal region. On T1-weighted axial imaging (repetition time (TR)/echo time (TE) = 480/11 ms), the mass appeared isointense to skeletal muscle (**Figure 1a**). T2-weighted axial images (TR/TE = 5,880/95 ms) (**Figure 1b**) and T2-weighted fat-suppressed axial images (TR/TE = 6,550/97 ms) (**Figure 1c**) demonstrated a slightly high-signal intensity (relative to the skeletal muscles) and did not exhibit a low-intensity region on T2-weighted fat-suppressed axial images, suggesting the presence of fat tissue. Diffusion-weighted imaging (DWI) with b = 800 s/mm^2^ displayed markedly increased signal intensity (**Figure 1d**). The apparent diffusion coefficient (ADC) map indicated restricted diffusion (**Figure 1e**). The lesion had a visible envelope and a cystic necrosis in the center (**Figures a and b**). The capsule displayed a pronounced low-signal intensity rim on T2-weighted axial images. Intravenous Gadolinium diethylenetriamine pentaacetate (Gd-DTPA) injection revealed moderate uniform enhancement in the solid part of the mass, compared to the uterine myometrium (**Figure 1f**). The right normal ovary was not identified, suggesting the mass likely originated from the right ovary. No abnormal signals were observed in the left adnexal region, and no enlarged pelvic lymph nodes were evident. The preoperative imaging diagnosis was a right ovarian thecoma, with differential diagnoses, including ovarian solid tumors like Brenner’s tumor and extra-ovarian tumors such as subplasmal or broad ligament leiomyoma. Based on the MRI findings and clinical presentation, surgery was recommended.

## Surgical and pathological findings

**Figure 2.**
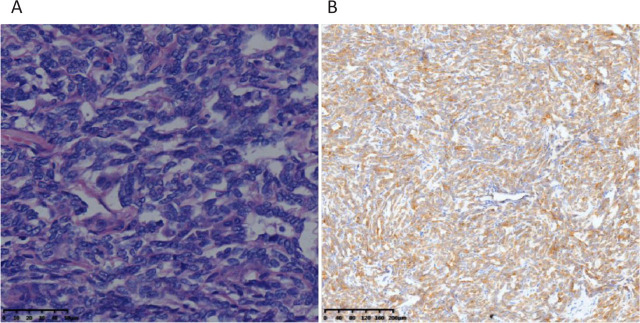
Epithelial tumor cells were arranged in packed strands with a slit or fern-leaf-like structure (HE ×400) **(A)**. Tumor cell cytoplasm was positive for a-inhibin (EnVision method ×400) **(B)**. HE, hematoxylin & eosin.

The right ovary exhibited enlargement, measuring approximately 12 cm in diameter, and displayed a vascular-rich, purplish-blue surface. The uterus was enlarged, resembling a gestational age of over 50 d, with a protrusion of approximately 4 cm × 3 cm near the cervix on the posterior uterine wall. The left adnexal region appeared normal in appearance, with no evident abnormalities within the abdominal cavity. Approximately 50 mL of yellowish ascitic fluid was observed. The bilateral adnexa and the uterus were surgically removed. The right ovarian tumor presented as a solid and brittle mass, measuring approximately 12 cm × 12 cm. Intraoperative frozen pathology suggested a potential malignancy, necessitating routine and immunohistochemical confirmation. Microscopic examination revealed a diffuse arrangement of epithelial tumor cells, forming a slit or fern-leaf-like structure with rare nuclear divisions (**Figure 2A**). The lumens were lined with a single layer of round nuclei featuring fine chromatin. Immunohistochemical staining indicated positive results for alpha-inhibin (**Figure 2B**), Calretinin (CR), CD10, pan cytokeratin (CK-P), cytokeratin (CK)19, Wilms tumor protein (WT-1), Des, P16, Melan-A, and Vim, along with approximately 20% Ki-67 nuclear positivity. Negative results were obtained for Pax-8, CK7, epithelial membrane antigen (EMA), androgen receptor (AR), estrogen receptor (ER), and progesterone receptor (PR). Based on the immunohistochemistry and hematoxylin and eosin (HE) morphology, the diagnosis was established as a Wolffian tumor originating from the right ovary. Postoperative intervention was not required, and the patient was monitored for 6 months, during which no recurrence or metastasis was observed.

## Discussion

Wolffian tumors originate from the remnants of the Wolffian duct, specifically the mesonephric duct. They may manifest within various remnants of the mesonephric duct, including the hilum of the ovary, fallopian tube tract, lateral aspect of the uterus, and the outer third of the vagina, with the broad ligament and fallopian tube tract being common sites of origin [2]. Typically, patients develop Wolffian tumors between the ages of 15 and 81, with an average onset at around 50 years [3]. This neoplasm is commonly observed in middle-aged women [3]. Tumor size can vary from 0.8 cm to 20 cm [4]. Clinical symptoms are often nonspecific, with approximately 53% of the patients being asymptomatic and diagnosed incidentally during gynecological examinations. Some patients may experience abdominal distension, pain, bloating, vaginal bleeding, or endocrine-related symptoms [2]. The site, age of onset, and clinical symptoms in this case align with those reported in the literature.

While most literature suggests that Wolffian tumors are benign, occasional malignancies have been reported, with approximately 10% of the cases demonstrating postoperative recurrence or metastasis [3, 4]. Malignancy is more likely in cases with a tumor diameter of ≥10 cm, increased anisotropy and nuclear schizotypy, invasion of adjacent structures, implantation, and metastasis. Complete surgical resection remains the primary treatment. Given the tumor’s malignant potential, preoperative diagnosis and assessment are of paramount importance.

MRI plays a crucial role in the preoperative diagnosis of pelvic tumors in women; however, Wolffian tumor-related imaging reports are scarce, with only 8 cases reported [5,6,7,8]. In 1999, Matsuki et al. [5] reported the first case of Wolffian tumor, characterized by an ovoid mass with well-defined borders. The tumor displayed iso-signal intensity on T1-weighted images, slightly high-signal intensity on T2-weighted fat-suppressed images, and a cystic area at the lesion periphery. The solid portion exhibited homogeneous enhancement following gadolinium-DTPA administration, while the cystic area showed no enhancement [5]. Subsequent reports by Sato et al. [6] and Sakai et al. [7] in 2012 and 2016, respectively, described MRI findings consistent with those observed in this case, including solid adnexal masses. Ito et al. [8] reviewed preoperative MRI images in 4 surgical cases of Wolffian tumor in 2021. The MR imaging findings in all the 9 cases are summarized in this study. The majority of tumors presented as round or oval, with 2 cases displaying local nodular features. In all, 5 of the 9 lesions were solid, while others were solid with cystic components. In all cases, T1-weighted images displayed signal intensities that were iso or slightly low in comparison to skeletal muscles, and T2-weighted images exhibited intermediate intensities. Additionally, all Wolffian tumors displayed markedly increased signals on DWI with restricted ADC in the solid components. Six of the 9 lesions presented a prominent low-signal intensity rim at the tumor’s edge, indicating the presence of a tumor envelope. Fat-suppressed contrast-enhanced T1-weighted images, available in 7 cases, revealed moderate enhancement in the solid part. No peritoneal implants or enlarged lymph nodes were detected in any of the cases, and no malignant MRI findings were observed. The MRI presentations of Wolffian tumors corresponded with the gross pathology in all cases [9].

Distinguishing Wolffian tumors from solid adnexal neoplasms such as subplasma or broad ligament leiomyoma, Brenner’s tumor, fibroma–thecoma group, adult granulosa cell tumors (GCTs), and endometrioid cancer is essential, based on the clinical features and the imaging findings. Additionally, other solid adnexal tumors include dysgerminomas and yolk sac tumors, which are more commonly encountered in children or young women, whereas Wolffian tumors are more prevalent in middle-aged women and, therefore, do not fall within the scope of the differential diagnosis. (1) Subplasma or broad ligament leiomyoma primarily consists of smooth muscle cells and fibrous connective tissue, resulting in low water and mucin content, which is reflected in low-signal intensity on T2-weighted images. In contrast, Wolffian tumors exhibit a slightly higher-signal intensity. Cell-rich leiomyomas can be challenging to differentiate as they predominantly consist of cell clusters with high water content, large cytosomes, and high-signal intensity on T2-weighted images. DWI can provide some discrimination, as cell-rich leiomyosarcomas may exhibit obscured diffusion restriction and often lack an intact envelope. (2) Brenner’s tumor is predominantly composed of epithelial cells and fibrous stroma. The rich fibrous stroma within the tumor often results in low-signal intensity on T_2_-weighted images, similar to muscle, and the presence of degenerative necrosis is infrequent. Notably, calcification is a common feature of Brenner’s tumor [10]. (3) The fibroma–thecoma group primarily consists of theca cells and typically displays high- or slightly high-signal intensity on T2-weighted images, presenting a similar imaging appearance to Wolffian tumors. However, a study has demonstrated that thecomas are generally homogenous solid masses with signal intensity similar to the myometrium on DWI, along with ADC values similar to leiomyomas [11]. In contrast, Wolffian tumors exhibit markedly high-signal intensity on DWI (4). Adult granulosa cell tumors (AGCTs) can present a range of MRI appearances, from entirely solid to multilocular cystic. The solid portion often displays high-signal intensity on DWI and low ADC values [12]. MRI findings for AGCTs and Wolffian tumors can overlap. However, AGCTs originate from granulosa cells, which produce estradiol, inhibin, and anti-Müllerian hormone (AMH) in premenopausal women. These substances serve as useful tumor markers for AGCTs. A combined measurement of serum inhibin B and AMH has been reported as highly sensitive (97%) and specific (81%) in detecting AGCTs [13]. (5) Endometrioid cancer typically presents as multilocular cystic masses with solid components. Numerous solid components often exist along the inner cystic surface and extend toward the center. This tumor may occasionally appear predominantly solid with a small central cavity, known as an internal slit [10]. This imaging feature can lead to confusion with Wolffian tumors. However, endometrioid cancer is often associated with endometriosis in the same ovary or elsewhere in the pelvis. Moreover, approximately 15%–30% of the cases are accompanied by synchronous endometrial hyperplasia or endometrial carcinoma [14].

## Conclusion

Wolffian tumor, originating from the middle renal duct remnants, is a rare and distinctive epithelial tumor, primarily characterized by its benign nature. MRI scans typically reveal Wolffian tumors as solid or cystic masses, with solid presentation being more common. These tumors manifest slightly low-signal intensity on T_1_WI and slightly high-signal intensity on T_2_WI. Notably, they display markedly high-signal intensity on DWI. Additionally, these masses often exhibit moderate and homogeneous enhancement, and they may feature a prominent low-signal intensity rim at their periphery, indicative of a tumor envelope. In some cases, cystic necrosis may also be observed. Despite the rarity of this condition, the MRI characteristics of Wolffian tumors are distinctive, and clinicians should consider this possibility when encountering similar lesions in clinical practice.
